# Supplementary material for: Event timing in human vision: Modulating factors and independent functions
Source: PLoS One. 2020 Aug 27;15(8):e0226122. doi: 10.1371/journal.pone.0226122 (PMC7451557; doi:10.1371/journal.pone.0226122)
Supplement: Attachment — Submitted filename: Response_to_Reviewers.docx [file pone.0226122.s002.docx]

Reviewer #2: I think the revised manuscript is generally improved with regards to my pointed out. However, I still have several minor questions and suggestions to the authors.

1. The distance was “11.1 degrees of visual angle between their centres” as stated in the Methods section.

In my previous question, I meant a distance between 2 stimuli could be crucial factor that affects the temporal resolution, although I understand this factor was fixed in the present study. I suggest that the authors add discussions about this as a possible spatial factor.

Response:

We agree with the reviewer and have added to the Discussion section the following discussion: “Further factors that should be taken into account by future studies relate to a systematic variation of spatial distances between stimuli (which were fixed in the current study), which might influence perceptual resolution.”

2. As stated in the Methods section: “The bar stimuli were faded in incrementally within 5 frames (8.33ms per frame) from 5.2 – 53.7 lux.”

My question was that then the brightness of the bar was kept at 53.7 lux till end of the trial?

Response:

We have added to the Methods section: “(and kept at that brightness until the response)”

3. The aim of the study was not to investigate (potentially) pathological processing of timing but deliver a set of factors influencing processing of stimulus timing in healthy individuals, which could serve as baseline for studies of time processing in psychopathologies.

I basically agree the reply and additional description of the authors. But, I’m still thinking that autistic traits is known to be distributed in healthy populations widely (i.e., autism spectrum), and then it could affect the results. I suggest adding short discussion about this as a possibilities individual factor.

Response:

We have added to the Discussion section: “In addition, individual trait factors such as autistic traits or impulsivity traits might arguably influence perception and response criteria and should be studied systematically in relation to event timing in human vision.”
